# Glucosidase inhibitory activity and antioxidant activity of flavonoid compound and triterpenoid compound from *Agrimonia Pilosa* Ledeb

**DOI:** 10.1186/1472-6882-14-12

**Published:** 2014-01-10

**Authors:** Xi Liu, Liancai Zhu, Jun Tan, Xuemei Zhou, Ling Xiao, Xian Yang, Bochu Wang

**Affiliations:** 1 Key Laboratory of Biorheological Science and Technology (Chongqing University), Ministry of Education, College of Bioengineering, Chongqing University, No. 174, Shapingba Main Street, Chongqing 400030, China; 2 School of Biological & Chemical engineering, Chongqing University of Education, Chongqing 400067, China; 3 College of Life Sciences, Chongqing Normal University, Chongqing 401331, China

**Keywords:** Type 2 diabetes mellitus, Flavonoid compound, Triterpenoid compound, Postprandial hyperglycemia, Oxidative stress

## Abstract

**Background:**

In Chinese traditional medicine, *Agrimonia pilosa* Ledeb (APL) exhibits great effect on treatment of type 2 diabetes mellitus (T2DM), however its mechanism is still unknown. Considering that T2DM are correlated with postprandial hyperglycemia and oxidative stress, we investigated the α-glucosidase inhibitory activity and the antioxidant activity of flavonoid compound (FC) and triterpenoid compound (TC) from APL.

**Methods:**

Entire plants of APL were extracted using 95% ethanol and 50% ethanol successively. The resulting extracts were partitioned and isolated by applying liquid chromatography using silica gel column and Sephadex LH 20 column to give FC and TC. The content of total flavonoids in FC and the content of total triterpenoids in TC were determined by using UV spectrophotometry. HPLC analysis was used to identify and quantify the monomeric compound in FC and TC. The α-glucosidase inhibitory activities were determined using the chromogenic method with p-nitrophenyl-α-D-glucopyranoside as substrate. Antioxidant activities were assessed through three kinds of radical scavenging assays (DPPH radical, ABTS radical and hydroxyl radical) & β-carotene-linoleic acid assay.

**Results:**

The results indicate FC is abundant of quercitrin, and hyperoside, and TC is abundant of 1β, 2β, 3β, 19α-tetrahydroxy-12-en-28-oic acid (265.2 mg/g) and corosolic acid (100.9 mg/g). The FC & the TC have strong α-glucosidase inhibitory activities with IC_50_ of 8.72 μg/mL and 3.67 μg/mL, respectively. We find that FC show competitive inhibition against α-glucosidase, while the TC exhibits noncompetitive inhibition. Furthermore, The FC exhibits significant radical scavenging activity with the EC_50_ values of 7.73 μg/mL, 3.64 μg/mL and 5.90 μg/mL on DPPH radical, hydroxyl radical and ABTS radical, respectively. The FC also shows moderate anti-lipid peroxidation activity with the IC_50_ values of 41.77 μg/mL on inhibiting β-carotene bleaching.

**Conclusion:**

These results imply that the FC and the TC could be responsible for the good clinical effects of APL on T2MD through targeting oxidative stress and postprandial hyperglycaemia. So APL may be good sources of natural antioxidants and α-glucosidase inhibitors exhibiting remarkable potential value for the therapy of T2DM.

## Background

Type II diabetes mellitus (T2DM) as an epidemic disease, associated with increased significant social and financial burden, is a cause of very high morbidity and mortality in the world [1]. It is well-known that the genesis and progression of T2DM is generally attributed to several factors including persistent hyperglycemia toxicity, oxidative damage and lipotoxicity, etc. [1]. A widely held notion is that postprandial hyperglycemia (PPHG) is a primary risk factor in the development of T2DM and its complications via multi-factorial mechanisms [2]. Meanwhile, increasing evidences suggest that oxidative stress (OS) is involved in the pathogenesis of T2DM and the development of diabetic complications through the mechanism of impairing oxidation reduction system, leading to β-cell failure and insulin resistance [3,4].

Despite great efforts that have been made to normalize blood glucose level in clinic practice, it is still a formidable challenge. Even more difficult is the control of PPHG [5]. Nowadays, the more attentions are paid to control PPHG based on the α-glucosidase, α-amylase, amylin analogues as targets [6]. Among them, most therapeutic approaches for controlling PPHG are the pharmacological inhibitors with greatest effect on α-glucosidase including acorbose, miglilol, emigitate, voglibiose, etc. [6,7]. However, the continuous use of those synthetic agents should be limited because those agents may cause side effects such as flatulence, abdominal cramp, vomiting, and diarrhea [8,9]. Numerous studies have been carried out to screen natural agents (active natural components and crude extracts) to inhibit α-glucosidase activity without or with fewer side effects [10]. On the other hand, overwhelming researches suggest that natural antioxidants may be used to reduce oxidative damage and decrease the occurrence of diabetic complications [11,12]. Therefore, it is a prospective strategy that PPHG and ROS are used as dual-target to screen the natural drugs to combat the multiple disorders of T2DM.

*Agrimonia pilosa* Ledeb (APL), a Chinese traditional medicinal plant of Rosaceae family, is widely used to treat blood, tumor, gastrointestinal, gynecological, genitourinary diseases in Chinese traditional medicine [13]. Especially, in the past several decades Chinese traditional medicine have shown the great effect of APL on T2DM in clinical practice. But it is unclear how APL acts on T2DM. Chemical composition studies reveal that the APL is abounded with polyphenols, terpenoids and coumarins etc. [14,15]. It has been reported that some flavonoids and terpenoids from medicinal plants have the inhibitory activities of α-glucosidase [16]. Moreover, the abundant flavonoids are mainly responsible for the antioxidant activities of many herbs [17]. Therefore, we speculated that the APL could combat T2DM through targeting PPHG and OS.

In this study, we isolated the flavonoid compound (FC) and the triterpenoid compound (TC) from APL, and evaluated their α-glucosidase inhibition activity and antioxidative activities. Meanwhile, the inhibitory effect on α-glucosidase of the compounds with the different ratio of the FC and the TC also was tested. Furthermore, the inhibition kinetics against α-glucosidase of the FC and the TC were studied.

## Methods

### Chemicals

Butylated hydroxyl toluene (BHT), gallic acid, β-carotene, linoleic acid, 1,1-diphenyl-2-picrylhydrazyl (DPPH·), ρ-nitrophenyl α-D-glucopyranoside(PNPG), 3,5-dinitro salicylic acid, soluble potato starch and 1-deoxyrojirimycine, α-Glucosidase (from Saccharomyces cerevisiae), HPLC grade methanol and acetonitrile were purchased from Sigma Chemical Co. (St. Louis, MO, USA). Folin-Ciocalteu reagent was obtained from E. Merck Co. (Darmstadt, Germany). Standards including oleanolic acid, ursolic acid, vitexin, rutin, hyperoside, luteolin-7-O-β-D-glucopyranoside, quercitrin, quercetin, luteolin, apigenin and kaempferol were obtained from the National Institutes for Food and Drug Control (Beijing, China). 1β, 2β, 3β, 19α-tetrahydroxy-12-en-28-oic acid, tormentic acid, maslinatic acid, corosolic acid and tiliroside are isolated and identified by ourselves in lab. All other reagents were analytical grade procured from indigenous manufacturers.

### Plant materials and preparation of the extract

**Figure 1:**
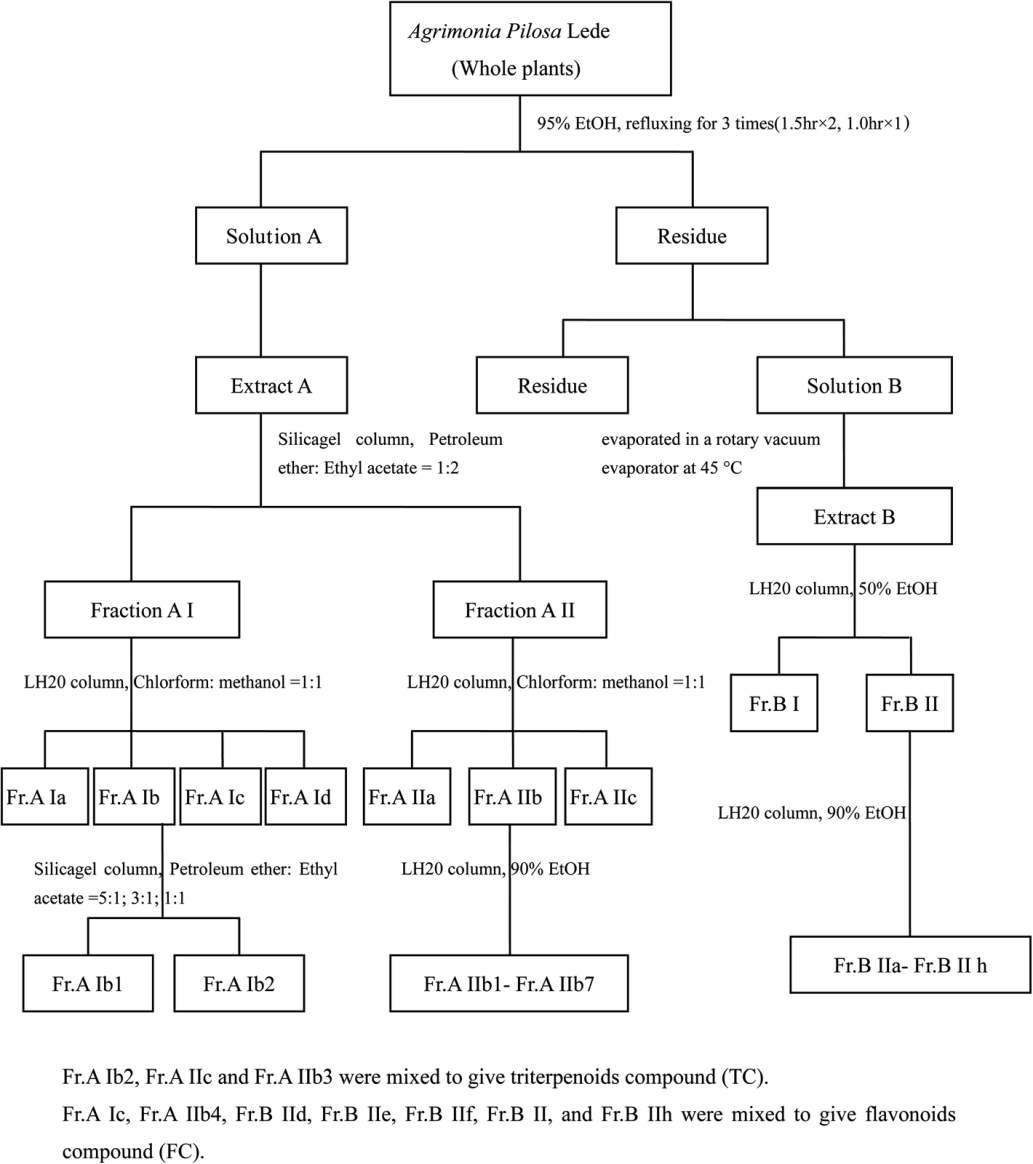
The collected procedure of flavonoid compound (FC) and triterpenoid compounds (TC).

The dried entire plants of APL were purchased from Western Medicine City (Chongqing, China) in 2011 and verified by Changhua Wang (Chongqing Academy of Chinese Materia Medica, China). Extracts were obtained as follows: In brief, the dried entire plants (2 kg) were chopped into small pieces (40 mesh) and soaked overnight in 40 L of 95% ethanol, then was under refluxing at 100°C for three times for 90 min, 90 min, 60 min, respectively. The suspension was filtered to give solution A. Then the residue was extracted by 40 L of 50% ethanol under refluxing as above condition to give solution B. The two filtrate solvents were evaporated in a rotary vacuum evaporator at 45°C and then lyophilized to give extract A and extract B. Then the extracts were separated using liquid column chromatography (see Figure 1). Finally, according to thin layer chromatography control with 10% sulfate in ethanol as color-developing agent, Fr.A Ib2, Fr.A IIc and Fr.A IIb3 were mixed to give triterpenoids compound (TC) and Fr.A Ic, Fr.A Ib4, Fr.B IId, Fr.B IIe, Fr.B IIf, Fr.B IIg, and Fr.B IIh were mixed to give flavonoid compound (FC). The TC powder (2.5646 g) and the FC powder (9.5375 g) were stored at 0–4°C.

### Determination of total flavonoids content

Total flavonoids content in FC was determined by using the aluminium chloride colorimetric method. A mixture of 0.5 mL sample, 100 μL 10% aluminum chloride, 100 μL 1 M potassium acetate and 2.3 mL distilled water were incubated at room temperature for 30 min. The absorbance was measured at 415 nm. Quercetrin as a standard was used to make the calibration curve. The estimation of total flavonoids in the extracts was carried out in triplicate and the results were averaged.

### Determination of total triterpenoids content

Total triterpenoid content in TC was determined by using UV spectrophotometry [18], with some modifications. Reference substance (0.8 mL) was moved in 10 mL volumetric flask, to evaporate ethanol in water bath at 85°C and then add 0.4 mL 5% vanillin-glacial acetic acid and 1.0 mL perchloric acid, respectively. The mixture was kept in water bath at 80°C for 20 min, cooled to room temperature in ice bath, and 5.0 mL glacial acetic acid was added. The solutions were mixed by manual shaking for 15–20 s and allowed to stand for 15 min at room temperature. Finally, the absorbance was measured at 765 nm. Ursolic as a standard was used to make the calibration curve. The estimation of total triterpenoid content in the extracts was carried out in triplicate and the results were averaged.

### HPLC analysis of flavonoids in FC

HPLC analysis was performed using Agilent1260 with UV detector, and chromatographic separations were conducted on a Welch ultimate XB-C18 column (4.6 × 250 mm, 5 μm). The solvent system was a gradient of mobile phase: soln. A: 0.1% phosphate in H_2_O; soln. B, acetonitrile. The following gradient was used: 0–30 min, 87% A; 30–50 min, 87% A to 80% A; 50–70 min, 80% A to 60% A; 70–80 min, 60% A to 87% A; 80-90 min, 87% A. Operating conditions were as follows: flow rate, 1.0 mL/min; column temp., 30°C; injection volume, 20 μL; UV detection at 350 nm.

### HPLC analysis of triterpenoids in TC

HPLC analysis was performed using Agilent 1260 with UV detector, and chromatographic separations were conducted on a Welch ultimate XB-C18 column (4.6 × 250 mm, 5 μm). The solvent system was a gradient of mobile phase: soln. A: 0.1% formic acid in H_2_O; soln. B: acetonitrile and methnol (2:1, v/v). The following gradient was used: 0–10 min, 25% A; 10–15 min, 25% A to 30% A; 15–30 min, 30%A. Operating conditions were as follows: flowrate, 1.0 mL/min; column temp., 30°C; injection volume, 20 μL; UV detection at 210 nm.

### Inhibition assay for α-glucosidase activity

The α-glucosidase inhibitory activities of TC and the FC were determined according to the chromogenic method with slight modifications [19]. The substrate solution p-nitrophenyl-α-D-glucopyranoside (pNPG, Sigma Chemical Company) was prepared with 0.2 M of Na-phosphate buffer (pH 6.8). The reaction mixture contained 10 μL of 0.02 U/μL α-glucosidase solution in 0.2 M Na-phosphate buffer (pH6.8), 10 μL of a sample in DMSO (or DMSO itself as blank control) with the concentration of 20.0, 50.0, 100.0, 200.0, 400.0, 500.0 μg/mL, respectively, 50 μL of Na-phosphate buffer (pH6.8), which were mixed and incubated at 37°C for 20 min. Then, 50 μL of 0.02 M PNPG was added, and the mixture was incubated at 37°C for another 30 min. Finally, the reaction was stopped by the addition of 100 μL 0.2 M Na_2_CO_3_ solutions. Amount of the p-nitrophenol released from PNP-glycoside was quantified on a 96 microplate spectrophotometer (Bio-Rad, USA) at 405 nm. The inhibitory rate of sample on α-glucosidase was calculated by the following formula.

$$\text{Inhibition}\;\left(\%\right)=\left[A_{\text{blank}}-\left(A_{\text{sample}}-A_{\text{background}}\right)\right]/A_{\mathrm{blank}}\times 100.$$

All the tests were run in triplicate. The IC_50_ values (concentration required to inhibit 50% enzyme activity) were calculated by applying logarithmic regression analysis from the mean inhibitory values.

### Kinetics of inhibition against α-glucosidase

Inhibition mode of the APL against α-glucosidase activities was measured with the increasing concentration of p-nitrophenyl-α-D-glucopyranoside as a substrate in the absence or presence of FC or TC at different concentrations. Inhibition type was determined by Lineweaver-Burk plot analysis of the data, which was calculated from the result according to Michaelis-Menten kinetics.

### 1,1-Diphenyl-2-picrylhydrazyl radical scavenging activity

Assay for the scavenging of stable free radical 1,1-diphenyl-2-picrylhydrazyl (DPPH) was carried out as reported earlier with some modifications [20]. Briefly, 100 μL sample in methanol was mixed with 1.9 mL of 0.1 mM DPPH in ethanol. The concentration of the tested samples in the mixture was 0.25, 0.5, 2.5, 5.0, 25.0, 50.0, 100.0 μg/mL, respectively. The reaction mixture was shaken well and incubated in dark at 37°C for 30 min and the absorbance was measured at 517 nm. The blanks contained all the reaction reagents except the extract or positive control substances. BHT was used as positive control. The percentage scavenging values were calculated from the absorbance of the blank (A_0_) and of the sample (A_S_) using the following equation:

$$\begin{array}{l} \mathrm{DPPH}\;\mathrm{radicals}\;\mathrm{scavenging}\;\mathrm{activity}\;\left(\%\right)\\ \;=\left(1–A_{S}/A_{0}\right)\times 100 \end{array}$$

Where A_S_ was the absorbance of the sample and A_0_ was the absorbance of the blank (without sample).

### Hydroxyl radical scavenging activity

The hydroxyl radical-scavenging assay was carried out using the method described by De Avellar and Jin with minor modifications [21]. 0.75 mM 1,10-phenanthroline and 0.75 mM FeSO_4_ were prepared in 0.05 M phosphate buffer (pH 7.4) and mixed thoroughly. 100 μL of sample in methanol at different concentration of 5.0, 10.0, 50.0, 100.0, 500.0, 1000.0, 2000.0 μg/mL respectively, and 880 μL of above solution (incubated at 37°C for 30 min) was dispensed to test tubes and 20 μL of H_2_O_2_ (0.01%) were added. The reaction mixture was incubated in dark at 37°C for 60 min, and measured at 536 nm. The hydroxyl radical scavenging activities were calculated using the following equation:

$$\begin{array}{l} \text{Hydroxyl}\;\text{radical}\;\text{scavenging}\;\text{activity}\;\left(\%\right)\\ \;=\left[\left(A_{S}–A_{c}\right)/\left(A_{0}–A_{c}\right)\right]\times 100 \end{array}$$

Where A_S_, absorbance of the sample; A_c_, absorbance of control solution containing 1,10-phenanthroline, FeSO_4_ and H_2_O_2_ without sample; A_0_, absorbance of blank solution containing 1,10-phenanthroline and FeSO_4_ without H_2_O_2_ and sample.

### ABTS radical scavenging assay

ABTS radical-scavenging activity was measured by modifying the method described by Re *et al.*[22]. ABTS was dissolved in de-ionized water to 7 mM concentration, and potassium persulphate was added to a concentration of 2.45 mM (final concentration). After well mixed, the reaction mixture was left to stand at room temperature overnight (12–16 h) in the dark before usage (Fresh stocks of ABTS solution were prepared every five days due to self-degradation of the radical). 200 μL sample in methanol at different concentrations of 1.25, 2.5, 12.5, 25.0, 125.0, 250.0, 500.0 μg/mL respectively was mixed with 0.3 mL ABTS^**∙**+^ solution and 0.5 mL distill water. The mixture was allowed to stand at room temperature for 2 min, and the absorbance at 745 nm was immediately recorded. Ascorbic acid served as a positive control. All the tests were performed in triplicate and the graph was plotted with the mean values. The assay was first carried out on the percentage of inhibition which calculated by the following formula:

$$\begin{array}{l} \mathrm{ABTS}\;\text{radical}-\text{scavenging}\;\text{activity}\;\left(\%\right)\\ \;=\left(1-A_{s}/A_{c}\right)\times 100 \end{array}$$

Where A_c_ was the absorbance of the blank (without sample) and A_s_ was the absorbance of the sample.

### Determination of the antilipid peroxidation activity with the β-carotene bleaching assay

The anti-lipid peroxidation activity of the sample was evaluated using β-carotene–linoleic acid model system [23,24]. 1 mL β-carotene (0.2 mg/mL) in chloroform was pipetted into a round-bottom flask (50 ml) containing 25 μL linoleic acid and 200 mg Tween-40. After the mixture was evaporated to remove chloroform under vacuum, 100 mL of distilled water saturated with oxygen was added by vigorous shaking to form an emulsion. 2400 μL of this emulsion was added into 100 μL of the sample in methanol with different concentrations. As soon as the mixture was added to each tube, the zero time absorbance was measured at 470 nm (A_S_^0^). The emulsion system was incubated for 2 h at 50°C (A_S_^t^). A blank without β-carotene was prepared for background subtraction (A_c_). BHT was used as a positive control. The test was carried out in triplicate. Results were calculated using the following equation:

$$\begin{array}{l} \text{Inhibiting}\;\text{activity}\;\mathrm{of}\;\beta -\text{carotene}\;\text{bleaching}\;\left(\%\right)\\ \;=\left[1–\left({A_{S}}^{0}–{A_{S}}^{t}\right)/\left({A_{c}}^{0}–{A_{c}}^{t}\right)\right]\times 100 \end{array}$$

### Statistical analysis

All tests were performed in triplicate. Results were expressed as the means ± SD (n = 3). The IC50 values were calculated from linear regression analysis. A paired-samples T test was used for the difference analysis between groups by using SPSS 19.0 software. Difference with a value of *p* < 0.01 were considered statistically significant.

## Results and discussion

Despite a great of efforts have been made in treatment of T2DM in clinic, poor effect accounted for a single target is still a major challenge. More and more researches suggested that the multi-target therapy combining with the control of postprandial hyperglycaemia and oxidative stress may become a promising therapy strategy because oxidative stress is mainly induced by postprandial hyperglycaemia [4]. There is increasing interest in screening of bioactive compounds from herbal plant with the ability to delay or prevent glucose absorption, and to reduce oxidative damage. APL, as Chinese traditional medicine, has been used to treat T2DM clinically for several decades, but its pharmaco-mechanism is not so clear. To address the mechanism at least to some degree, both α-glucosidase inhibiting activity and antioxidant activity of the FC and the TC from APL were studied. Many traditionally medicinal plants and natural products have been tested for their inhibition of α-glucosidase activities and anti-diabetic potential [10], but few researches about APL have been reported.

**Figure 2:**
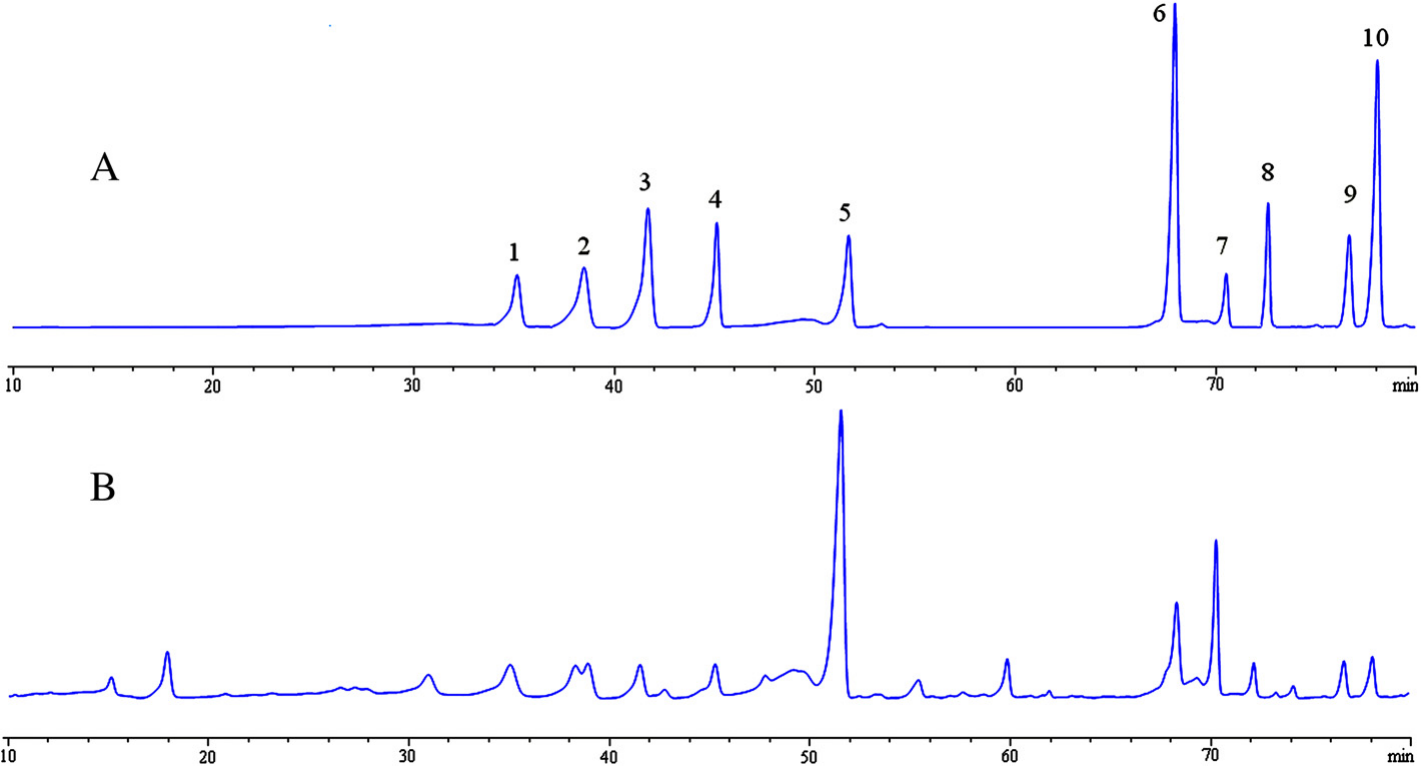
**HPLC chromatogram of flavonoid compositions in flavonoid compound (FC). (A)** standard flavonoids; **(B)** FC. Peaks: 1: vitexin; 2: rutin; 3: hyperoside; 4: luteolin-7-O-β-D-glucopyranoside; 5: quercitrin; 6: quercetin; 7: tiliroside; 8: luteolin; 9: apigenin; 10: kaempferol.

Based on the theory of the active components alignment, the FC and the TC were isolated from APL. The total flavonoids content in FC is 316.53 ± 6.37 mg/g by using the aluminum chloride colorimetric method with quercetrin as a standard (*y* = 0.01718*c* – 0.00556, *R*^2^ =0.9994; *x* was the concentration of the extract while *y* was the absorbance of the flavonoids). By HPLC analysis, 10 flavonoids were identified and quantified: vitexin, rutin, hyperoside, luteolin-7-O-β-D-glucopyranoside, quercitrin, quercetin, tiliroside, luteolin, apigenin and kaempferol (Table 1). Chromatographic profiles of flavonoids composition of FC are shown in Figure 2. We found that quercitrin, tiliroside and vitexin are abundant flavonoids which make up 64.3% of the 10 flavonoids.

**Table 1:** The contents of flavonoids in flavonoid compound (FC)

**Compounds**	**Contents (mg/g)**
Vitexin	24.2 ± 1.2
Rutin	10.8 ± 0.4
Hyperoside	9.8 ± 0.5
Luteolin-7-O-β-D-glucopyranoside	8.4 ± 0.8
Quercitrin	73.6 ± 2.6
Quercetin	13.9 ± 0.8
Tiliroside	31.2 ± 0.9
Luteolin	14.7 ± 1.1
Apigenin	11.3 ± 0.4
Kaempferol	2.7 ± 0.5

Note: Each value represents the mean ± S.D. (n = 3).

**Figure 3:**
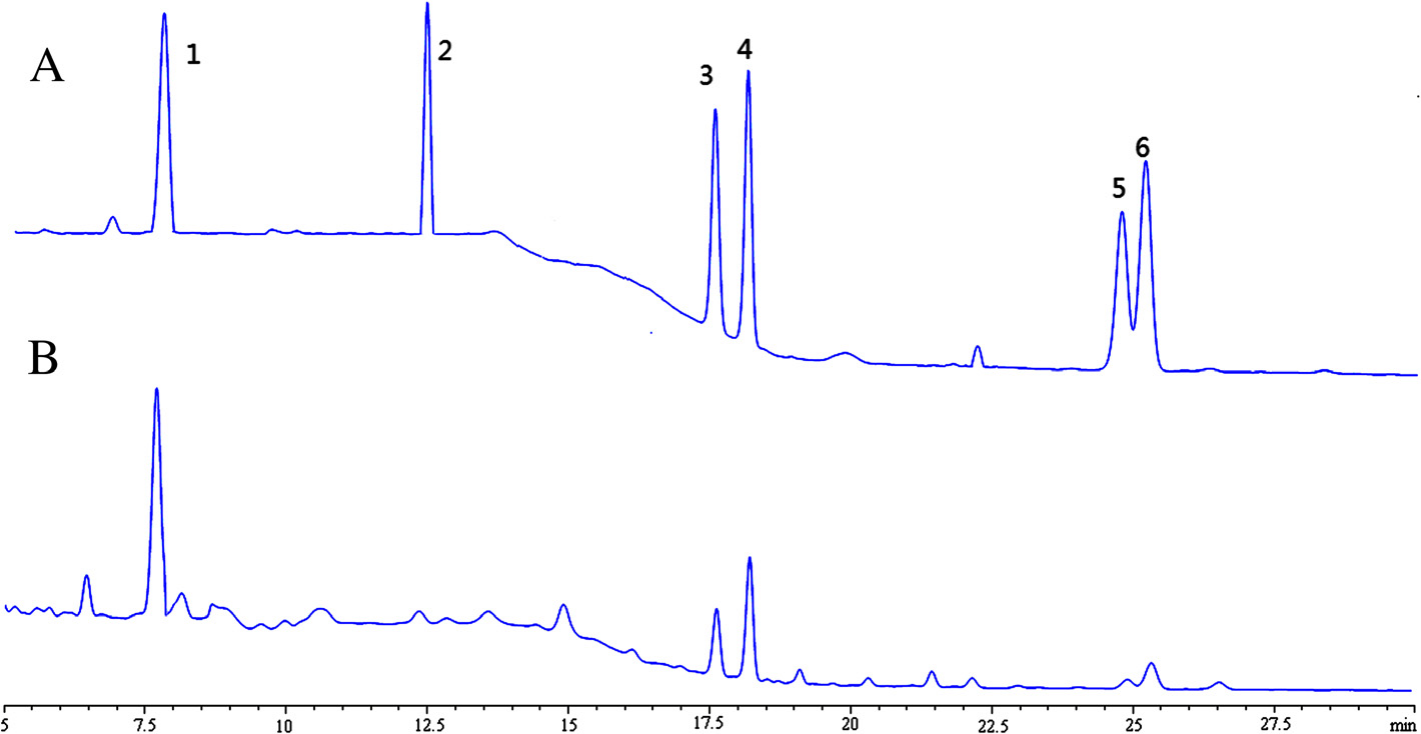
**HPLC chromatogram of triterpenoid compositions in triterpenoid compound (TC). (A)** standard triterpenoids; **(B)** TC. Peaks: 1: 1β, 2β, 3β, 19α-tetrahydroxy-12-en-28-oic acid; 2: tormentic acid; 3: maslinatic acid; 4: corosolic acid; 5: oleanolic acid; 6: ursolic acid.

The total triterpenoids content in TC is 415.97 ± 5.15 mg/g with ursolic as a standard (*y* = 0.05584*x* – 0.06093, *R*^2^ = 0.99949; *x* was the concentration of extract while *y* was the absorbance of triterpenoids). And 6 triterpenoids were identified and quantified by using HPLC analysis: 1β, 2β, 3β, 19α-tetrahydroxy-12-en-28-oic acid, tormentic acid, maslinatic acid, corosolic acid, oleanolic acid and ursolic acid (Table 2). Chromatographic profiles of triterpenoids composition of TC are shown in Figure 3. 1β, 2β, 3β, 19α-tetrahydroxy-12-en-28-oic acid is the most predominant triterpenoid in TC, contributing 265.2 mg/g. The followings are corosolic acid (100.9 mg/g), maslinatic acid (53.7 mg/g), ursolic acid (28.2 mg/g), tormentic acid (22.2 mg/g), oleanolic acid (6.7 mg/g). The contents of these six triterpenoids in TC add up to 476.9 mg/g.

**Table 2:** The contents of triterpenoids in triterpenoid compounds (TC)

**Compounds**	**Contents (mg/g)**
Oleanolic acid	6.7 ± 0.4
Ursolic acid	28.2 ± 0.4
Maslinatic acid	53.7 ± 0.5
Corosolic acid	100.9 ± 7.5
Tormentic acid	22.2 ± 1.7
1β, 2β, 3β, 19α-tetrahydroxy-12-en-28-oic acid	265.2 ± 2.0

Note: Each value represents the mean ± S.D. (n = 3).

### α-glucosidase inhibition activity

**Figure 4:**
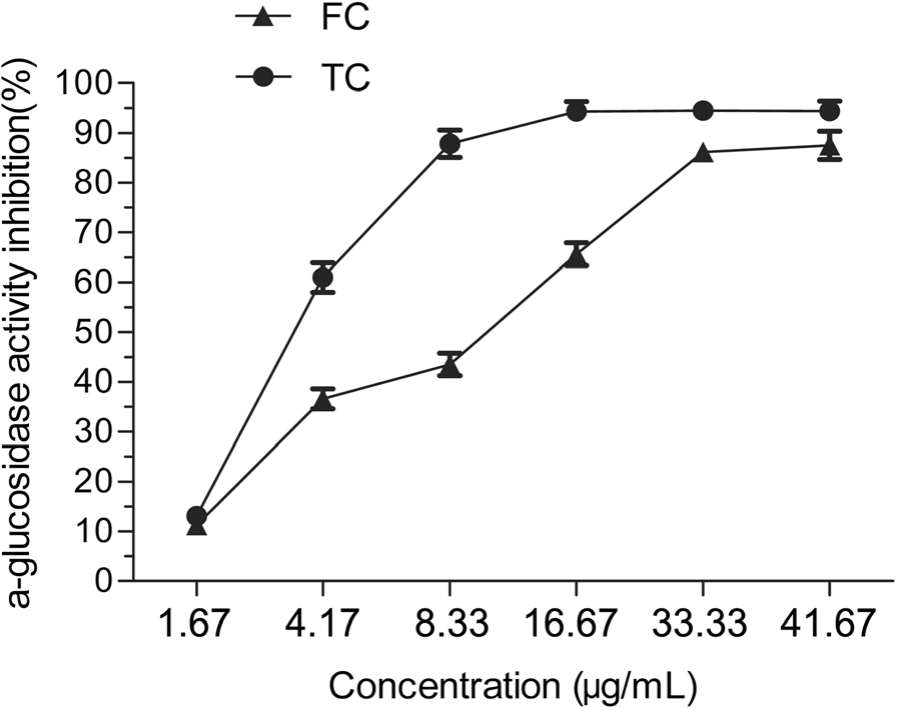
**Inhibitory activities of flavonoid compound (FC) and triterpenoid compound (TC) from *Agrimonia pilosa *** **Ledeb** ***. *** **against α-glucosidase.**

**Figure 5:**
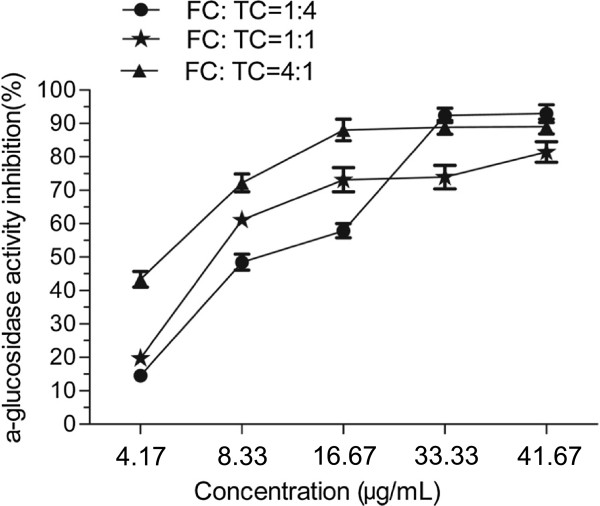
Inhibitory activities of the three complexes of flavonoid compound (FC) and triterpenoid compound (TC) with mass ratio of 4:1, 1:1 and 1:4 against α-glucosidase.

**Figure 6:**
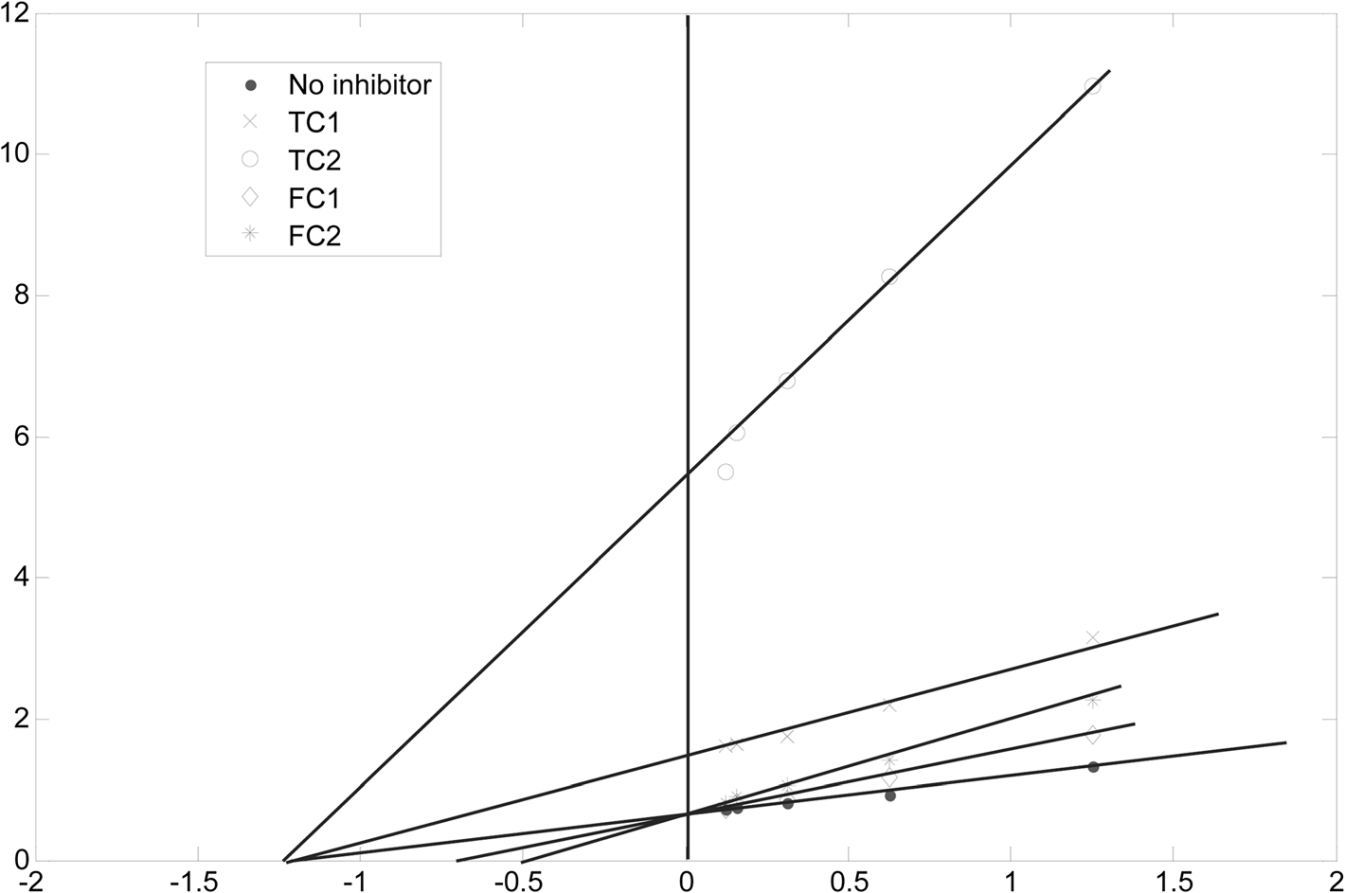
**Lineweaver-Burk plot for the inhibition mode of flavonoid compound (FC) and triterpenoid compound (TC) against α-glucosidase.** No inhibitor: absence of sample as a control; TC1: 8ug/mL; TC2: 16ug/mL; FC1: 8ug/mL; FC2: 16ug/mL.

Most importantly therapeutic approaches for decreasing PPHG were to prevent absorption of glucose by inhibition of α-glucosidase. So the inhibitory effects of the FC and the TC from APL against α-glucosidase were studied (Figure 4). And the IC_50_ (50% inhibitory concentration) values were analyzed by SPSS software. Both the FC and the TC could inhibit the activity of α-glucosidase in a dose-dependent manner at the concentrations of 1.67–41.67 μg/mL. The maximum inhibition is found to be 94.5% and 87.5% at the 41.67 μg/mL of the FC and the TC, respectively. Compared with the FC (IC_50_ = 8.72 μg/mL), the TC exhibits the stronger efficiency with the IC_50_ of 3.67 μg/mL. The activities of three complexes with mass ratio of FC and TC as 4:1, 1:1 and 1:4 were also determined. The data in Figure 5 show that the complex with the mass ratio of FC and TC as 4:1 exhibits the best inhibition activity against α-glucosidase among these three complexes. The IC_50_ values of three complexes with mass ratio of FC and TC as 4:1, 1:1 and 1:4 are 3.92, 10.39 and 9.39 μg/mL, respectively. Furthermore, the studies of enzyme kinetics (see Figure 6) demonstrated that the FC exhibits competitive inhibition against α-glucosidase, while TC exhibits noncompetitive inhibition.

Previously some plant extracts have been reported to inhibit α-glucosidase activity, such as the ethanol exract of *Andrographis paniculata* (IC_50_ = 17.20 mg/mL) [25], the ethanol extracts of *Mangifera indica* bark (IC_50_ = 314 μg/mL) [26], and the butanol extract of *Acosmium panamense* (IC_50_ = 109 μg/mL) [27]. Compared with these reported natural extracts, the FC and the TC from APL showed excellent α-glucosidase inhibitory activities. In addition, some natural products could exhibit different inhibition modes against α-glucosidase, such as the methanol fraction of Bitter melon in an uncompetitive manner with an IC_50_ value of 2.60 mg/mL [28]. Our results also show that the FC and the TC from APL inhibit α-glucosidase in different modes, i.e. the FC in competitive mode and the TC in noncompetitive inhibition mode. In addition, it was reported that corosolic acid from Lagerstroemia apeciosa leaves exhibit a nocompetitive mode with an IC_50_ value of 3.53 μg/mL [29]. And corosolic acid with the content of 100.9 mg/g also is the second abundant triterpenoid in the TC (Table 2). Furthermore, the inhibition activities against α-glucosidase of the complexes with different mass ratio of FC and TC were assayed based on their different inhibitory modes. The result reveals that the combination with mass ratio of FC and TC as 4:1 has superiority inhibition activity against α-glucosidase to the other complexes with mass ratio of FC and TC as 1:4 and 1:1 at the concentration range of 4.17- 33.33 μg/mL. But when the concentration is greater than 33.33 μg/mL, the complexes (FC: TC = 1:4, g/g) has better α-glucosidase inhibition activity than others. Based on the results mentioned above, we could propose that, at the low concentration, the competitive inhibition is dominant, but the noncompetitive inhibition has a little advantage at the high concentration. So we think that the APL has a potential treating effect on T2MD, which is attributed partly to the α-glucosidase inhibition activities of the FC and the TC for the control of the postprandial hyperglycaemia.

### *In vitro* antioxidant activity

In order to obtain the credible conclusion, four assays including DPPH scavenging assay, hydroxyl radical scavenging assay, ABTS radical scavenging assay and β-carotene-linoleic acid assay were employed to evaluate antioxidant activities of the FC and the TC.

**Figure 7:**
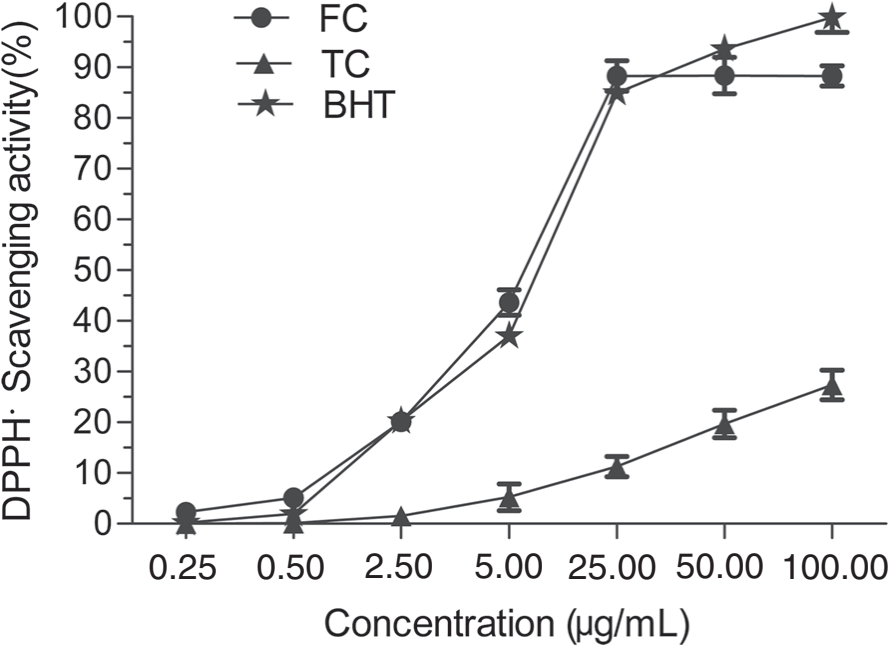
**DPPH radical scavenging activities of flavonoid compound (FC) and triterpenoid compound (TC).** Each value represents the mean ± S.D. (n = 3).

**Figure 8:**
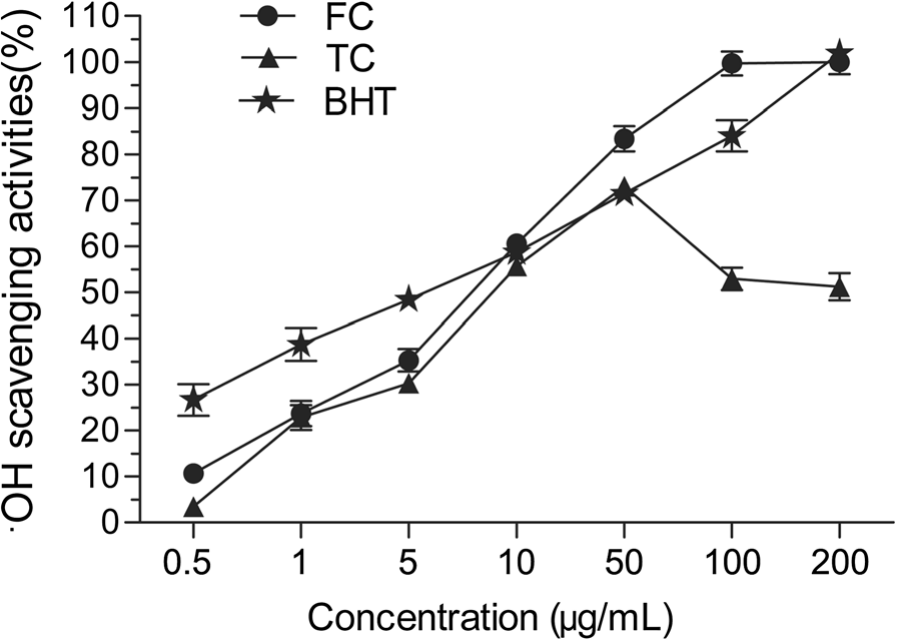
**OH radical scavenging activities of flavonoid compound (FC) and triterpenoid compound (TC).** Each value represents the mean ± S.D. (n = 3).

**Figure 9:**
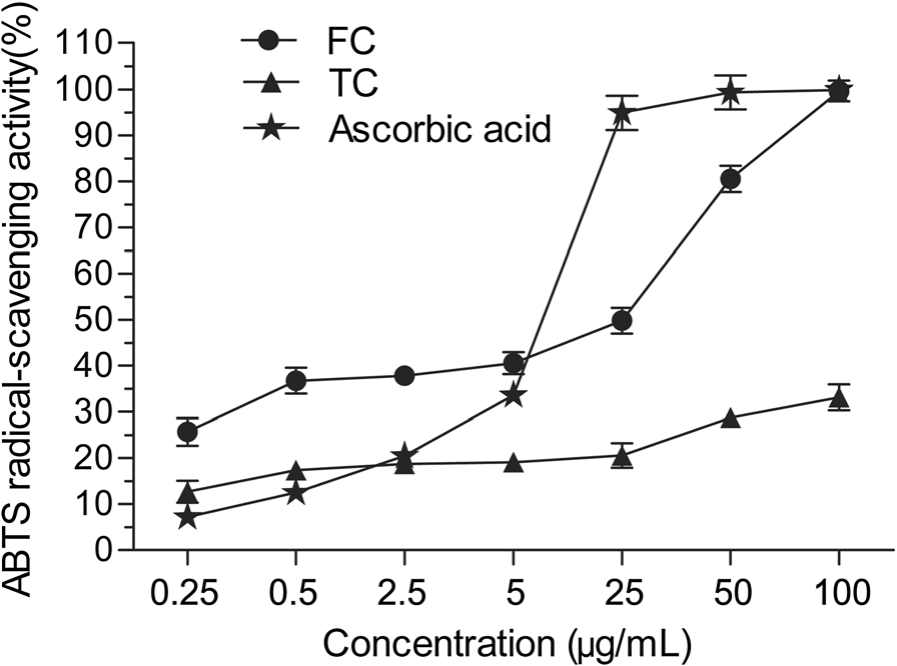
**ABTS radical scavenging activities of flavonoid compound (FC) and triterpenoid compound (TC).** Each value represents the mean ± S.D. (n = 3).

The free radical scavenging activities of the FC and the TC were evaluated using DPPH scavenging assay, hydroxyl radical scavenging assay and ABTS radical scavenging assay. As shown in Figures 7, 8 and 9, the FC has significant radical scavenging activity in a dose-dependent manner, which is comparable with BHT. The EC_50_ values of the FC on DPPH radical, hydroxyl radical and ABTS radical are 7.7 μg/mL, 3.6 μg/mL and 5.9 μg/mL, respectively (Table 3). The EC_50_ values of BHT which was used as positive control on DPPH radical and hydroxyl radical are 7.7 μg/mL and 3.1 μg/mL, respectively. And the EC_50_ values of the ascorbic acid used as positive control on ABTS is 2.5 μg/mL. Compared with the FC, the TC has weaker radical scavenging activity with EC_50_ values on DPPH radical, hydroxyl radical and ABTS radical of >100.0 μg/mL, 35.2 μg/mL and >100.0 μg/mL, respectively (Table 3).

**Table 3:** **The EC**_**50 **_**values and IC**_**50 **_**values of flavonoid compound (FC) and triterpenoid compounds (TC) from *agrimonia pilosa *** **ledeb**

**Compounds**	**Radical scavenging (EC**_**50**_**, μg/mL)**			**β-carotene bleaching (IC**_**50**_**, μg/mL)**
	**DPPH·**	**·OH**	**ABTS**	
FC	7.7 ± 0.4	3.6 ± 0.2	5.9 ± 0.4*	41.8 ± 1.0*
TC	>100.0	35.2 ± 0.3*	>100.0	98.5 ± 1.2*
BHT	7.7 ± 0.2	3.1 ± 0.1	-	1.8 ± 0.2
Ascorbicacid	-	-	2.5 ± 0.1	-

Note: An asterisk (*) indicates a significant difference at the p < 0.01 level *vs* positive control.

Each value represents the mean ± S.D. (n = 3).

**Figure 10:**
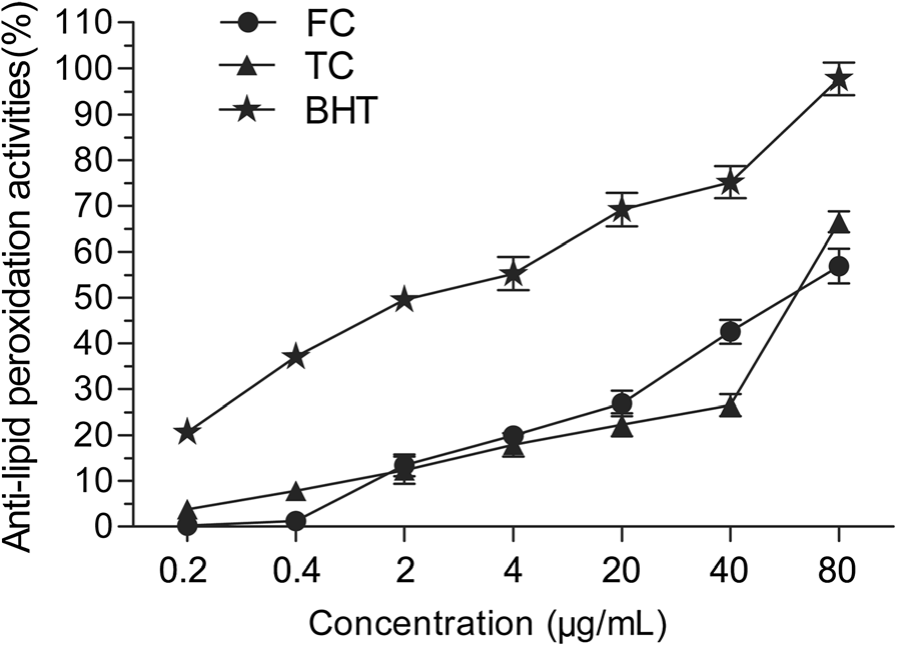
**Anti-lipid peroxidation activities in the β-Carotene-linoleic acid system of flavonoid compound (FC) and triterpenoid compound (TC) from *Agrimonia pilosa *Ledeb.** Each value represents the mean ± S.D. (n = 3).

The anti-lipid peroxidation activity of the FC and the TC were determined by using the β-carotene-linoleic acid system and the results were shown in Figure 10. All of the tested samples have inhibition activity on lipid peroxidation in a dose-dependent manner (0.2 μg/mL-80.0 μg/mL). BHT shows an excellent inhibitory activity (IC_50_ = 1.8 μg/mL), while the IC_50_ values of the FC and the TC are 41.8 μg/mL and 98.5 μg/mL, respectively. It is demonstrated that FC has moderate anti-lipid peroxidation activity.

Among the quantified 10 flavonoids in FC, 6 flavonoids including quercetin, luteolin, rutin, hyperoside, luteolin-7-O-β-D-glucopyranoside and quercitrin have excellent scavenging activities on radical due to the o-catechol group (3′, 4′–OH) in B ring [30]. These 6 flavonoids making up 65.4% of the quantified 10 flavonoids could be responsible for the FC’s significant scavenging activities on DPPH radical, hydroxyl radical and ABTS radical. Radicals induced by postprandial hyperglycaemia or other reasons [4,31], such as superoxide anion radical and hydroxyl radical, have the very high reactivity which enables them to react with a wide range of molecules, such as protein, lipids, and nucleotides leading to occurrence and development of a variety of diseases including T2DM [3,32]. The remarkable scavenging activities of the FC on radical imply that the FC could be responsible for the good clinical effects of APL on T2MD targeting oxidative stress.

## Conclusions

In conclusion, our findings suggest that the FC and the TC could be responsible for the good clinical effects of APL on T2MD through targeting oxidative stress and postprandial hyperglycaemia. So APL may be good sources of natural antioxidants and α-glucosidase inhibitors exhibiting remarkable potential application in the therapy of T2DM. Further investigations should be taken to illustrate the pharmaco-mechanism deeply and to isolate the active components.

## Competing interests

The authors declare that they have no competing interests.

## Authors’ contributions

XL carried out the experimentation, acquisition and analysis of data and drafting of the manuscript. LZ conceived, designed, supervised the study and revised the manuscript. JT provided technical support and advice in the experiments and revised the manuscript. XZ carried out the acquisition of the *Agrimonia pilosa* Ledeb extracts. LX & XY carried out the HPLC analysis of FC and TC. BW supervised the study and helped in drafting and revision of manuscript. All authors have read and approved the final manuscript.

## Pre-publication history

The pre-publication history for this paper can be accessed here:

http://www.biomedcentral.com/1472-6882/14/12/prepub
